# Overcoming chemoresistance in esophageal cancer with synergistic strategies

**DOI:** 10.3389/fimmu.2026.1784325

**Published:** 2026-06-03

**Authors:** Chunxiao Gao, Ting Zhang, Guangyuan Li, Weishan Zhang, Honglin Chen, Diefei Li, Jixuan Xu, Mengxue Li, Ruihong Wang, Yamin Xing, Jian Zhan, Zhentai Ren, Xia Xue, Wulong Liang, Xiangdong Sun, Xiaojuan Zhang, Xinfeng Liang, Hongqiao Zhan, Zisen Zhang, Xiufeng Chu

**Affiliations:** 1 Zhengzhou Key Laboratory of Lipid Metabolism and Precision Diagnosis & Treatment in Oncology, Oncology Department, the Fifth Affiliated Hospital of Zhengzhou University, Zhengzhou, China; 2 State Key Laboratory of Metabolic Dysregulation & Prevention and Treatment of Esophageal Cancer, Tianjian Laboratory of Advanced Biomedical Sciences, Zhengzhou, China; 3 Marshall B. J. Medical Center, The Fifth Affiliated Hospital of Zhengzhou University, Zhengzhou, China; 4 Department of Gastrointestinal & Thyroid Surgery, The Fifth Affiliated Hospital of Zhengzhou University, Zhengzhou, Henan, China; 5 Department of Respiratory and Critical Care Medicine, The Fifth Affiliated Hospital of Zhengzhou University, Zhengzhou, Henan, China; 6 Henan International Joint Laboratory of Glioma Metabolism and Microenvironment Research, The Fifth Affiliated Hospital of Zhengzhou University, Zhengzhou, Henan, China; 7 Scientific Research and Discipline Management Office, The Fifth Affiliated Hospital of Zhengzhou University, Zhengzhou, Henan, China

**Keywords:** chemoresistance, esophageal cancer, immunotherapy, targeted therapy, tumor microenvironment

## Abstract

Esophageal cancer is a globally prevalent malignancy with poor clinical outcomes. Chemotherapy remains an effective strategy for esophageal cancer. However, its effectiveness is substantially limited by chemoresistance, which arises from both tumor cell adaptation and changes in the surrounding microenvironment. In light of these challenges, combination strategies show great promise for overcoming chemoresistance. Notably, the integration of chemotherapy with PD-1/PD-L1 blockade has demonstrated substantial survival benefits across multiple phase III clinical trials. This review provides a comprehensive overview of the mechanisms underlying chemotherapy resistance in esophageal cancer. More importantly, we summarize the current advances in chemotherapy-based combination therapies, as well as nanoparticle−based delivery systems and antibody−drug conjugates. These advances reinforce the potential for developing more effective therapeutic strategies.

## 1 Introduction

Since the initial efforts to use chemotherapeutic agents such as bleomycin, mitomycin C, and 5-fluorouracil (5-FU) for esophageal cancer treatment in the late 1960s, researchers have conducted extensive research to improve chemotherapy strategies (1, 2). For recurrent or metastatic esophageal squamous cell carcinoma (ESCC) and esophageal adenocarcinoma (EAC), first-line systemic therapy is commonly based on a fluoropyrimidine (5-FU or capecitabine) plus a platinum agent (3). Currently, chemotherapy has evolved its role from its initial use as a palliative attempt in advanced-stage patients to a cornerstone across the entire spectrum of esophageal cancer management. For locally advanced patients, neoadjuvant chemoradiotherapy or chemotherapy alone has become a standard strategy to enhance surgical resectability and improve survival rates. For those with recurrent or metastatic disease, chemotherapy remains the core palliative approach for alleviating symptoms and prolonging survival (3, 4). Furthermore, chemotherapy remains a fundamental component of current combination therapies (5–7).

Chemoresistance is a major barrier to the efficacy of chemotherapy. For patients with esophageal cancer who receive chemotherapy alone, the reported objective response rate remains limited, ranging from 22.7% to 57.8% across studies (5, 8). Intrinsic resistance derives from cellular adaptations, including upregulation of drug efflux pumps, enhanced DNA repair, and loss of cell cycle checkpoints (9, 10). Extrinsic resistance is imposed by the tumor microenvironment (TME), where factors such as hypoxia and aberrant vasculature foster a protective niche for tumor cells (11). From a temporal perspective, chemotherapy resistance can pre-exist, emerge during the malignant transformation of esophageal epithelial cells before treatment, or develop under the selective pressure of drug exposure during therapy. Overcoming chemotherapy resistance is a critical challenge for improving patient outcomes (12–16). This review examines the current understanding of the mechanisms underlying chemotherapy resistance in esophageal cancer and explores promising therapeutic strategies to overcome it, with particular emphasis on rational combinations of chemotherapy with targeted therapy and immunotherapy.

## 2 Chemoresistance

### 2.1 Intrinsic chemoresistance

#### 2.1.1 Drug efflux and metabolism

Reducing the intracellular accumulation of chemotherapeutic agents through drug efflux represents a critical mechanism by which tumor cells evade cytotoxicity (17–19). In ESCC, tumor cells upregulate the membrane-associated scaffolding protein Caveolin-1, which in turn enhances the expression of P-glycoprotein (P−gp) and multidrug resistance-associated protein 1 (MRP1) (20), both of which are ATP-binding cassette (ABC) transporters actively pump out drugs such as 5−FU, paclitaxel, and platinum−based agents from cells (21–23).

In addition, Phase I cytochrome P450 (CYP) enzymes and Phase II conjugating enzymes are closely associated with chemoresistance in esophageal cancer (24, 25). For example, CYP3A4 metabolizes paclitaxel into less cytotoxic derivatives (26, 27). GST-π (GSTP1) catalyzes the conjugation of platinum-based agents with glutathione (GSH) and neutralizes their cytotoxicity (28, 29). Additionally, metallothioneins (MTs) also play a key role in chemoresistance by binding to and reducing the efficacy of platinum-based agents (30, 31).

Efforts have been made to develop P-gp inhibitors to overcome drug efflux. The gut-specific inhibitor Encequidar (HM30181A) has completed a Phase III trial in breast cancer and demonstrated favorable safety and efficacy (NCT06835400). Meanwhile, another P-gp inhibitor, MB07133, is being evaluated in a Phase II trial for hepatocellular carcinoma (NCT00073736) (**Supplementary Table 1**). Although dedicated clinical trials of P-gp (or GST) inhibitors are currently lacking in esophageal cancer, the promising results from other malignancies suggest their potential applicability to overcoming chemoresistance in this setting (20, 32).

#### 2.1.2 DNA damage and repair

Platinum-based agents bind to DNA to form intra- or inter-strand crosslinks. This structural distortion induces severe DNA damage (10, 33). 5-Fluorouracil (5-FU) is an antimetabolite drug that mediates DNA and RNA damage through inhibition of thymidylate synthase and incorporation of its metabolites into DNA and RNA (34). Although DNA damage is intended to trigger tumor cell apoptosis, tumor cells counteract this effect through the DNA damage response (DDR) system (33, 35, 36). Key repair pathways include homologous recombination repair (HRR), non-homologous end joining (NHEJ), and nucleotide excision repair (NER). In esophageal cancer, elevated expression of crucial DDR proteins, such as RAD51 (in HRR) (37), DNA-PK and Ku70/80 (in NHEJ) (38, 39), and ERCC1 (in NER) (40), is closely associated with resistance to chemotherapy. Given the pivotal role of DDR in chemoresistance, inhibitors against key DDR regulators are under active development (41, 42). A prime example is the use of PARP inhibitors (e.g., Olaparib, Niraparib) (43). In tumor cells with a pre-existing HRR defect, PARP inhibition further disrupts the repair of single-strand breaks (SSBs). This dual impairment of both major repair pathways leads to an accumulation of lethal DNA damage, a mechanism known as synthetic lethality (41, 42). In esophageal cancer, combining PARP inhibitors with DNA-damaging chemotherapy is being actively explored. The rationale is to use chemotherapy to create DNA damage while employing a PARP inhibitor to block its repair, thereby synergistically enhancing tumor cell killing. This approach is particularly effective for esophageal cancer harboring homologous recombination deficiency. Corresponding clinical trials are underway, including studies evaluating Olaparib (NCT01460888) and Niraparib (NCT03840967) in esophageal/gastroesophageal cancers (**Supplementary Table 1**).

#### 2.1.3 Cell-cycle bypass and apoptosis evasion

Chemotherapeutic agents employ diverse mechanisms to exert cytotoxicity on tumor cells, but they converge on inducing cell cycle arrest and apoptosis (44). Esophageal cancer has evolved multiple strategies to counteract this effect (10). For instance, the Notch1 pathway is aberrantly activated to upregulate the activity of the Cyclin D1/CDK4/6 complex and accelerate the G1-to-S phase transition. This effectively shortens the therapeutic window for S-phase-specific agents such as 5-FU, allowing tumor cells to escape their cytotoxic effects (45). Additionally, tumor cells overexpress APE1 to upregulate RAD51 and facilitate rapid transit through the G2/M checkpoint, thereby diminishing the efficacy of DNA-damaging agents like cisplatin (37). Furthermore, the upregulated BubR1 (BUB1B) enables cells to undergo mitotic slippage and proceed into the next cell cycle, thereby bypassing the mitotic arrest induced by microtubule-stabilizing agents such as paclitaxel (46, 47).

To overcome cell cycle-related chemoresistance, CDK4/6 inhibitors have been developed. Among them, Palbociclib (Ibrance), Ribociclib (Kisqali), and Abemaciclib (Verzenio) are already approved for clinical use in breast cancer. In esophageal cancer, PD-0332991 and SHR6390 have demonstrated efficacy in suppressing tumor growth in preclinical experiments (48, 49). Moreover, a phase II clinical trial led by the Abramson Cancer Center to evaluate Palbociclib in advanced esophageal and gastroesophageal cancers (NCT01037790) is underway (**Supplementary Table 1**).

Remodeling of apoptotic signaling pathways is also observed in esophageal cancer. For example, in paclitaxel-resistant esophageal cancer models, anti-apoptotic proteins BCL-2, BCL-xL, and MCL-1 are upregulated, whereas pro-apoptotic proteins Bax, Bak, and Bim are suppressed (50, 51). Mechanistically, CtBP2 suppresses cisplatin-induced apoptosis by reducing the levels of p53, Bax, and cleaved caspase-3, while simultaneously increasing the level of BCL-2 (52). Additionally, RACK1 enhances chemoresistance to cisplatin and 5-FU by activating the PI3K/AKT pathway and upregulating BCL-2 expression (50).

To overcome apoptosis-related chemoresistance, BCL-2 inhibitor AT-101 has been found to enhance the cytotoxic effects of chemotherapeutic agents by promoting apoptosis in esophageal cancer (53). In addition, BCL-2/BCL-xL inhibitor TW37, when combined with the P-gp inhibitor verapamil, effectively increases the sensitivity of esophageal cancer to paclitaxel (32). Furthermore, the frequent loss of p53 function in esophageal cancer enables tumor cells to evade apoptosis induction (54, 55). Restoring p53 function has therefore emerged as another strategy to re-sensitize tumors. For instance, APR-246 (also known as eprenetapopt, a first-in-class small-molecule reactivator of mutant p53 currently under clinical investigation) (56), in combination with 5-FU, has demonstrated synergistic induction of apoptosis in preclinical esophageal cancer models (57, 58). Notably, this combination is also being evaluated clinically (NCT02999893) (**Supplementary Table 1**).

#### 2.1.4 Cancer stem cells

CSCs possess the fundamental properties of stem cells, including self-renewal, multilineage differentiation, and the ability to enter a dormant state (59, 60). These characteristics are crucial for driving tumor heterogeneity and conferring resistance to chemotherapy (61–63). ALDH1, a key regulator of CSC formation and maintenance through pathways such as HIF-1α/VEGF and Wnt/β-catenin, is highly expressed in esophageal cancer and other solid tumors (64). Furthermore, it has been shown that Notch signaling drives the stemness and tumorigenicity of esophageal adenocarcinoma (65). The Hippo coactivator YAP1 upregulates SOX9 and endows esophageal cancer cells with stem-like properties (66). circMALAT1 maintains CSC properties by regulating MSI2 ubiquitination and its subsequent degradation in ESCC (67). In addition, Shiozaki et al. found that TRPV2 and SLC12A2 cooperatively regulate intra- and extracellular Ca^2+^ and Cl^−^ concentrations to confer esophageal CSCs with resistance to cisplatin (68).

When CSCs enter a quiescent or dormant state, they are intrinsically insensitive to cell cycle-dependent chemotherapeutic agents (69–72). Multiple mechanisms are involved in this regulation of CSC dormancy. For example, redox metabolic pathways are reprogrammed to maintain the dormant status of CSCs during chemotherapy (73). In addition, the cell cycle-dependent inhibitor p27 suppresses cyclin-CDK activity to prevent CSCs from re-entering the proliferative cycle, while p21 reinforces G0/G1 arrest to keep CSCs in a non-proliferative state (74). In esophageal cancer, Wei et al. found that QSOX1 increases reactive oxygen species (ROS) levels and activates the PD-L1 signaling pathway, thereby promoting immune evasion of CSCs (75). In addition, PGC-1α, a key regulator of mitochondrial energy metabolism, is closely associated with stemness maintenance in various gastrointestinal malignancies (76–79). Marrone et al. reported that CSCs upregulate PGC-1α to enhance OXPHOS metabolism and maintain their quiescent state under environmental stress in pancreatic cancer (71).

Current research into CSC-targeted strategies has yielded several promising findings. For instance, blockade of the Notch signaling pathway with γ-secretase inhibitors has been shown to reduce the CSC population and enhance chemosensitivity in models of esophageal adenocarcinoma (80). Additionally, the drug disulfiram, when combined with copper ions, has demonstrated the ability to suppress CSC properties and reverse resistance to microtubule inhibitors in preclinical models of non-small cell lung cancer and head and neck cancer (81). However, to date, no CSC-targeted clinical trials in esophageal cancer have been registered or reported.

### 2.2 Tumor microenvironment

Beyond direct cytotoxic effects, part of the efficacy of chemotherapy depends on the induction of anti-tumor immunity via immunogenic cell death (ICD). In this context, an immunosuppressive TME can attenuate chemotherapy efficacy by impairing antigen presentation and limiting the activation and cytotoxic function of effector T cells, thereby weakening this immune-dependent component of treatment response and contributing to functional chemoresistance (82–85). For example, doxorubicin-induced immunogenic cell death of melanoma cells leads to enhanced dendritic cell-mediated antigen presentation and intratumoral CD8^+^ T cell anti-tumor immunity (86). Anthracycline-based chemotherapy triggers ATP release from fibrosarcoma cells and promotes the recruitment of myeloid antigen-presenting cells into the tumor microenvironment (87). However, esophageal cancer is typically characterized as an immunologically “cold” tumor, in which a profoundly immunosuppressive microenvironment severely limits the potential for chemotherapy-induced anti-tumor immunity (88). In this situation, even if tumor cells are naturally sensitive to cytotoxic drugs, a lack of effective anti-tumor immunity may still lead to poor tumor clearance (89–91). This suppression is primarily driven by the following key factors:

#### 2.2.1 Fibroblasts and immune cells in the TME

The TME is a highly heterogeneous and continuously evolving ecosystem that plays a decisive role in tumor initiation, progression, and therapeutic response (92). The interactions among different cell populations within the TME not only shape the biological behavior of tumors but also greatly influence their sensitivity to chemotherapy (93, 94).

**Cancer-Associated Fibroblasts (CAFs)** are one of the major non-tumor cell types contributing to the immunosuppressive TME (95, 96). CAFs have multiple origins and are mainly thought to arise from the continuous activation of resident fibroblasts in tissues surrounding the tumor in response to tumor-derived signals (97). Compared with normal fibroblasts, CAFs show stronger contractile properties and increased extracellular matrix production and are typically maintained in an activated, myofibroblast-like state (98). These characteristics form the biological basis of their function. CAFs modulate drug response through multiple mechanisms. In esophageal cancer, CAF-secreted IL-6 acts on tumor cells to activate the STAT3/NF-κB signaling pathway, thereby inducing resistance to cisplatin (99, 100). Specific CAF subpopulations secrete metabolites, such as phosphatidylcholine and glycerophosphocholine, that act as ligands to bind G protein-coupled receptors on tumor cells, thereby activating the JAK2/STAT3 pathway, and promoting resistance to FAK inhibitors (96). Moreover, Liang et al. found that fibroblasts in lymph node metastatic lesions (LN-Fbs) secrete PI16 to modulate cisplatin-sensitivity by inhibiting p38 and JNK signaling pathways in tumor cells (101).

**Immune Cells:** CD8^+^ T cells serve as the primary effector cells mediating antitumor immunity. In esophageal cancer, CD8^+^ T cells are not only numerically scarce but also functionally impaired owing to the elevated expression of immune checkpoint molecules, including PD-1, LAG-3, and TIM-3 (102, 103). In addition, M2-type tumor-associated macrophages (TAMs) suppress dendritic cells and effector T cells through the secretion of IL-10 and TGF-β (104). Regulatory T cells (Tregs) secrete IL-10 and TGF-β to suppress the functions of dendritic cells and effector T cells. Myeloid-derived suppressor cells (MDSCs) inhibit effector T cells through IL-6/STAT3-driven upregulation of arginase-1 and the production of reactive oxygen species (ROS) and inducible nitric oxide synthase (iNOS) (105, 106). For a more comprehensive overview of how different cells shape the tumor microenvironment, readers are referred to dedicated reviews on the TME (107–109).

A remaining important question is how the interaction networks between CAFs and various immune cells collectively establish the final immunosuppressive tumor microenvironment, and which components within these networks represent the most critical therapeutic targets. These issues still require further investigation.

#### 2.2.2 Hypoxia and angiogenesis

Hypoxia is commonly observed in various solid tumors (110, 111), including esophageal cancer (112, 113). HIF-1α, the central regulator of the cellular hypoxic response, is less efficiently hydroxylated under hypoxic conditions, thereby escaping normal degradation (111, 114). Accumulated HIF-1α upregulates TMTC3 expression and enhances VEGFA expression through the IMPDH2/GTP-Rho GTPase-STAT3 axis, thereby further promoting angiogenesis (115). Notably, the neovessels formed in this context are often structurally disorganized, abnormally permeable, and functionally inefficient, resulting in uneven local perfusion, elevated interstitial fluid pressure, and persistent hypoxia. These changes not only reduce the effective concentration of chemotherapeutic agents reaching the tumor parenchyma, but also provide a favorable microenvironment for the survival of tumor cells (116, 117). Given these mechanistic insights, combining VEGFR- and HIF-1α-targeted agents with chemotherapy represents a promising strategy to enhance therapeutic response (118–120). In addition to impairing drug delivery, hypoxia can directly promote chemoresistance through metabolic reprogramming. For example, Fang et al. showed that, in ESCC, hypoxia promotes cisplatin resistance through IGF1R-mediated arginine/proline metabolic reprogramming, whereas the combination of the IGF1R inhibitor linsitinib and cisplatin exerts a synergistic sensitizing effect (121).

It is noteworthy that these reported mechanisms of chemotherapy resistance in esophageal cancer have also been widely documented in other malignancies. These findings suggest that many resistance mechanisms may reflect shared biological processes across tumor types rather than mechanisms unique to esophageal cancer alone. A strict distinction between “shared” and “esophageal cancer-specific” mechanisms of chemoresistance would require direct comparative evidence across different malignancies under comparable experimental or clinical conditions. However, such evidence remains limited, and therefore, it is currently difficult to draw definitive conclusions regarding which mechanisms are truly specific to esophageal cancer.

## 3 Chemotherapy-based combinations to overcome chemoresistance in clinical practice

To overcome chemoresistance in esophageal cancer, the cornerstone of combining chemotherapy with other modalities is to achieve synergistic enhancement of anti-tumor effects (122–124). In this multimodal therapeutic landscape, chemotherapy remains central for eliminating rapidly proliferating tumor cells (125). Radiation therapy provides local tumor control by delivering high-dose radiation to tumor lesions. Targeted therapy blocks the key molecular pathways essential for tumor formation and development (123). Importantly, all three modalities are capable of inducing immunogenic cell death (126–128), thereby generating a more immunogenic tumor microenvironment and creating favorable conditions for subsequent immunotherapy (129). As a “game-changer,” immunotherapy functions by overcoming immunosuppressive signals in the tumor microenvironment and reactivating anti-tumor immune responses (103). Beyond local control, the resulting immune activation can induce systemic “abscopal effects” (130, 131), and may provide long-term control of tumor progression (103). This integrated strategy holds great promise for achieving synergistic enhancement and overcoming chemoresistance in esophageal cancer (123).

### 3.1 Chemotherapy combined with PD-1 inhibitors

In advanced/metastatic ESCC, the most significant advances in improving objective response rates (ORR), progression-free survival (PFS), and overall survival (OS) have been achieved by combining chemotherapy with PD-1 inhibitors. For example, the KEYNOTE-590 phase III clinical trial demonstrated that adding pembrolizumab to chemotherapy (platinum plus 5-FU) significantly improved survival in patients with advanced esophageal carcinoma, with particularly pronounced benefit in those with high PD-L1 CPS expression (132, 133). A similar result was observed in the CheckMate 648 study, which evaluated the combination of nivolumab and chemotherapy (platinum plus 5-FU) (134). Furthermore, sintilimab combined with chemotherapy, as well as camrelizumab combined with chemotherapy, has also shown markedly improved response rates in large phase III studies (132–138). These encouraging results have established PD-1/PD-L1 blockade combined with chemotherapy as a new standard for treating advanced or metastatic ESCC. The key outcomes of these pivotal clinical trials are summarized in **Table 1**.

**Table 1:** Clinical trials for the combination of chemotherapy and immunotherapy.

Trail	Year	Registration	Sample size	EC Type	Phase	Region	Experimental group	Control group	mOS(m)	mPFS(m)	ORR(%)	mDFS(m)
Qin et al. (135)	2024	ChiCTR2000040034	391	LA-ESCC	III	China	CAMRE + CHEMO (TP) CAMRE + CHEMO (nab-TP)	CHEMO (TP)	NA	NA	71% vs 69% vs 47%	NA
Kato et al. (137)	2024	NCT03189719	141	LA/mESCC	III	Japan	PENBRO + CHEMO(CF)	PLACEBO + CHEMO(CF)	17.7 vs 11.7	6.3 vs 6.0	56.8% vs 38.8%	NA
Lu et al. (136)	2022	NCT03748134	659	LA/mESCC	III	Global	SINTILI + CHEMO(CP/CF) (CP 93%)	Placebo + CHEMO(CP/CF) (CP 93%)	17.2 vs 13.6 (PD-L1 CPS ≥10) 16.7 vs 12.5	8.3 vs 6.4 (PD-L1 CPS ≥10) 7.2 vs 5.7	68% vs 49% (PD-L1 CPS ≥10) 66% vs 45%	NA
Doki et al. (134)	2022	CheckMate648 NCT03143153	970	Adv ESCC	III	Global	NIVO + CHEMO(CF)	CHEMO(CF)	13.2 vs 10.7 (PD-L1 CPS ≥1) 13.7 vs 10.2	6.9 vs 4.4 (PD-L1 CPS ≥1) 5.8 vs 5.6	53% vs 20% (PD-L1 CPS ≥1) 47% vs 27%	NA
Cao et al. (132)	2021	NCT03189719	749	LA/mESCC	III	Asia	PENBRO + CHEMO(CF)	PLACEBO + CHEMO(CF)	12.4 vs 9.8	6.3 vs 5.8	45.0% vs 29.3%	NA
Janjigian et al.	2021	NCT03691090	596	Adv/mESCC	III	China	CAMRE + CHEMO(CP)	PLACEBO + CHEMO(CP)	15.3 vs 12.0	6.9 vs 5.6	72.1% vs 62.1%	NA
Kojima et al.	2020	NCT02564263	628	Adv/mESCC	III	Global	PENBRO(2L)	CHEMO (DTX/PTX/IRI)	9.3 vs 6.7 (PD-L1 CPS ≥10)	2.6 vs 3.0 (PD-L1 CPS ≥10)	21.5 % vs 6.1 % (PD-L1 CPS ≥10)	NA

mOS, median overall survival; mPFS, median progression-free survival; ORR, objective response rate; mDFS, median disease-free survival; ESCC, esophageal cancer; PEMBRO, pembrolizumab; CAMRE, camrelizumab; NIVO, nivolumab; SINTILI, sintilimab; CHEMO, chemotherapy; CF, 5-fluorouracil and cisplatin; CP, cisplatin plus paclitaxel; TP, paclitaxel plus cisplatin; nab-TP, nab-paclitaxel plus cisplatin; CAP, capecitabine; DTX, docetaxel; PTX, paclitaxel; IRI, irinotecan; NA, not assessed.

### 3.2 Chemotherapy combined with targeted therapy

The exploration of this combination strategy in esophageal cancer is also actively underway. The VEGFR-2 inhibitor ramucirumab, when combined with chemotherapy, has shown significant survival benefits in patients with ESCC (phase II trial) and EAC (phase II/III trials) (139, 140). In addition, in a phase III clinical trial involving patients with HER2-positive advanced gastric or gastroesophageal junction cancer, adding trastuzumab to chemotherapy significantly improved median overall survival (141). Another promising innovation is development of antibody-drug conjugates (ADCs), which represent an innovative targeted chemotherapeutic approach. By combining the specificity of monoclonal antibodies with the potent cytotoxicity of chemotherapeutic agents, ADCs offer the potential to reduce off-target toxicity while enhancing tumor selectivity (142–145). Although preliminary clinical trials of ADCs in esophageal and other solid tumors are ongoing, their efficacy requires further validation through larger-scale clinical studies (146). The key outcomes of these pivotal clinical trials are summarized in **Table 2**.

**Table 2:** Clinical trials for the combination of chemotherapy and targeted therapy.

Clinical trial	Year	Registration	Sample size	EC type	Phase	Region	Experimental group	Control group	mOS(m)	mPFS(m)	ORR(%)	mDFS(m)
Scheck et al. (139)	2024	NCT03762564	21	Adv/mESCC	II	Germany	RAM + CHEMO (PTX)	CHEMO (PTX)	12.1 vs 9.2	3.8 vs 3.5	18.2% vs 20.0%	NA
Goetze et al. (140)	2023	NCT02661971	180	GEJ AC	II/III	Germany、Italy	RAM + CHEMO (FLOT)	CHEMO (FLOT)	46 vs 45	32 vs 21	82% vs 96%	NA
Xu et al.	2021	NCT02898077	440	Adv GAC/GEJ AC	III	Global	RAM + CHEMO (PTX)	PLACEBO + CHEMO (PTX)	8.71 vs 7.92	4.14 vs 3.15	26.5% vs 21.9%	NA
Fuchs et al.	2019	NCT02314117	645	Adv GC/ GEJ AC	III	Global	RAM + CHEMO (CF)	PLACEBO + CHEMO (CF)	11.2 vs 10.7	5.7 vs 5.4	41.1% vs 36.4%	81.9% vs 76.5%
Yoshikawa et al.	2019	NCT02539225	189	GC/ GEJ AC	II	Asia	RAM + CHEMO (S-1/OX)	PLACEBO + CHEMO (S-1/OX)	14.65 vs 14.26	6.34 vs 6.74	45.2% vs 46.4%	NA
Yoon et al.	2016	NCT01246960	168	Adv/mESCC, GEJ, Gastric AC (≈ 48% ES, 52% GEJ/Gastric)	II	US	RAM + CHEMO (mFOLFOX6)	PLACEBO + CHEMO (mFOLFOX6)	11.7 vs 11.5	6.4 vs 6.7	45.2% vs 46.4%	NA
Wilke et al.	2014	NCT01170663	665	Adv/Met ES, GEJ, Gastric AC (≈ 48% ES, 52% GEJ/Gastric)	III	Global	RAM + CHEMO (PTX)	PLACEBO + CHEMO (PTX)	9.6 vs 7.4	4.4 vs 2.9	28% vs 16%	NA

mOS, median overall survival; mPFS, median progression-free survival; ORR, objective response rate; mDFS, median disease-free survival; ESCC, esophageal squamous cell carcinoma; GEJ, gastroesophageal junction; AC, adenocarcinoma; RAM, ramucirumab; CHEMO, chemotherapy; PTX, paclitaxel; FLOT, 5-fluorouracil, leucovorin, oxaliplatin, and docetaxel; CF, 5-fluorouracil and cisplatin; OX, oxaliplatin;mFOLFOX6, 5-fluorouracil, leucovorin, and oxaliplatin; NA, not assessed.

### 3.3 Chemotherapy combined with radiotherapy

Radiotherapy exerts its anti-tumor effects primarily by inducing DNA double-strand breaks and interfering with the DNA damage response (DDR) through ionizing radiation. By delivering a “second strike,” radiotherapy acts as an effective partner in enhancing the cytotoxic effects of chemotherapeutic agents, particularly platinum-based drugs and 5-FU (147, 148). Increasing evidence indicates that radiotherapy achieves synergistic enhancement of therapeutic efficacy across different combination treatment strategies and disease stages in esophageal cancer (149). Yang et al. showed that neoadjuvant chemoradiotherapy, when compared to neoadjuvant chemotherapy alone, yields higher median overall survival (mOS) and overall response rate (ORR) in patients with locally advanced esophageal squamous cell carcinoma (LA-ESCC) (150, 151). In addition, neoadjuvant chemoradiotherapy-based integrated strategies, either combined with surgery or with immune checkpoint inhibitors, were associated with higher pathological complete response (pCR) rates (152) in LA-ESCC. Furthermore, in patients with advanced ESCC, definitive chemoradiotherapy was associated with longer mOS and mPFS than chemotherapy alone (153). The key outcomes of these pivotal clinical trials are summarized in **Table 3**.

**Table 3:** Clinical trials for the combination of chemotherapy and radiotherapy.

Clinical Trial	Year	Sample size	Conditions	Region	Experimental Group	Control Group	mOS(m)	mPFS(m)	ORR(%)	mDFS(m)	pCR
Yang et al. (150)	2024	206	LA-ESCC	China	Surgery after nCRT + ICIs	Surgery after nCT + ICIs	81.70 vs 84.42	NA	79.2% vs 73.4%	80.47% vs 83.21%	52.1%vs 32.3%
Yang et al. (151)	2024	1,162	LA-ESCC	Global	Surgery after nCRT	Surgery after nCT	66 vs 53	54.5 vs 32.4	NA	NA	32.6% vs 3.6%
Chen et al. (96)	2024	409	IVB-stage ESCC	China	Chemotherapy + PD-1 Inhibitor + Radiotherapy	Chemotherapy + PD-1 Inhibitor	24.9 vs 14.6	14.2 vs 10.6	NA	NA	NA

mOS, median overall survival; mPFS, median progression-free survival; ORR, objective response rate; mDFS, median disease-free survival; ESCC, esophageal cancer; nCRT, neoadjuvant chemoradiotherapy; nCT, neoadjuvant chemotherapy; ICIs, Immune Checkpoint Inhibitors; NA, not assessed.

## 4 Conclusion

**Figure 1:**
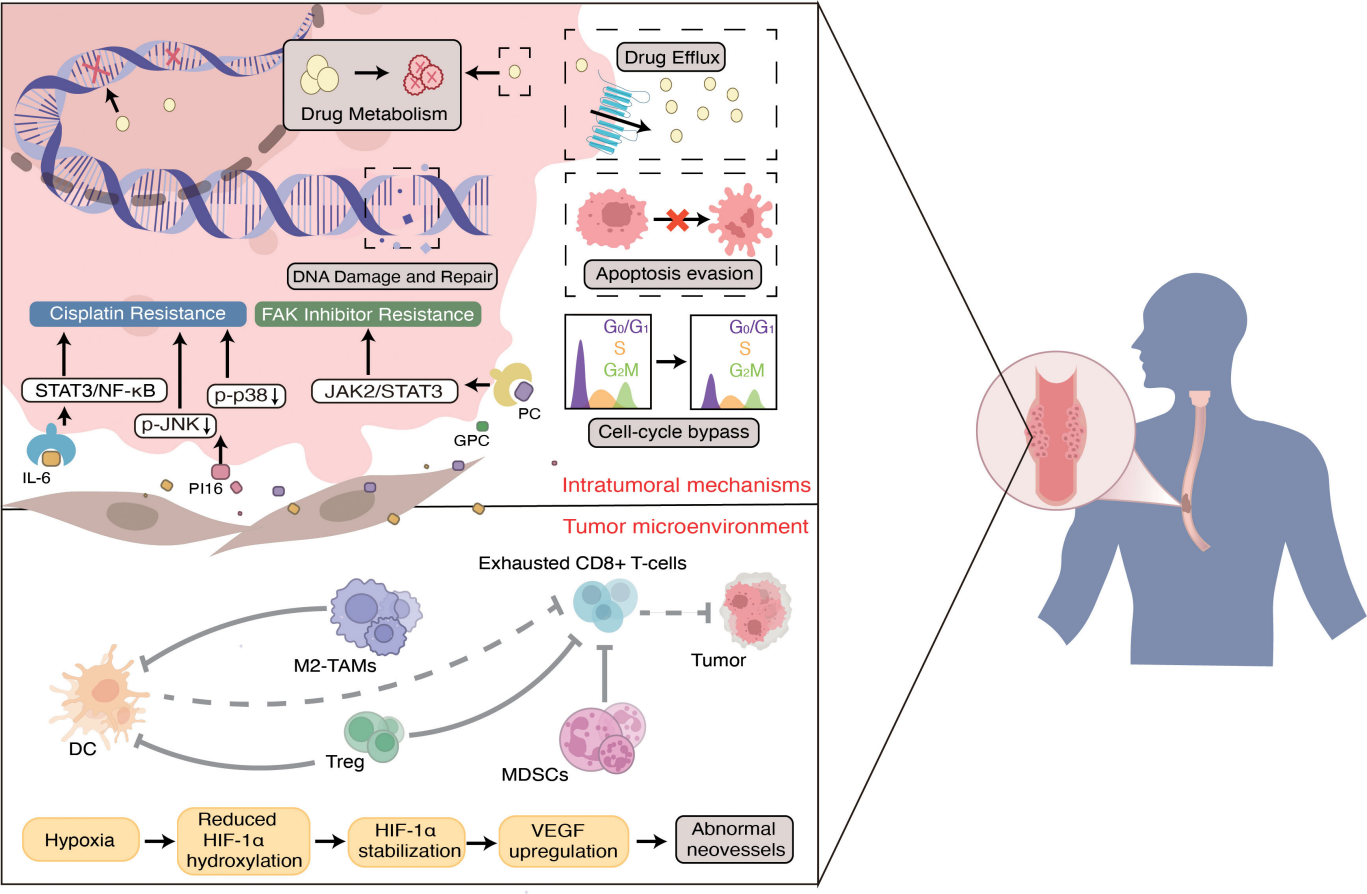
Integrated pathways driving chemoresistance in esophageal cancer. Cancer cells acquire chemoresistance through multiple mechanisms, including altered drug metabolism, increased drug efflux resulting in insufficient intracellular drug concentrations, enhanced DNA damage repair, dysregulated cell-cycle checkpoint control, and apoptosis evasion—collectively representing key intracellular adaptive responses. Additionally, the tumor microenvironment, characterized by hypoxia-induced aberrant angiogenesis and the presence of immunosuppressive cells such as Tregs and MDSCs, further facilitates the development and maintenance of drug resistance. These features highlight the multifactorial, dynamic, and highly adaptive nature of tumor resistance mechanisms. Abbreviation: PC, phosphatidylcholine; GPC, glycerophosphocholine; PI16, peptidase inhibitor 16; IL-6, interleukin-6; DC, dendritic cell; Treg, regulatory T cell; MDSCs, myeloid-derived suppressor cells; M2-TAMs, M2-type tumor-associated macrophages.

The development of chemoresistance in esophageal cancer is a complex and multifactorial process, driven by diverse mechanisms (**Figure 1**). To address this challenge, the integration of chemotherapy with other modalities, particularly PD-1 blockade, has ushered esophageal cancer treatment into a new era. In addition, emerging drug-delivery platforms, including nanoparticles and antibody-drug conjugates, allow for highly selective delivery, thus reducing off-target toxicity and overcoming pharmacokinetic drug resistance (145).

However, tumors continuously evolve and may evade combined chemotherapy and PD-1 blockade through mechanisms, such as loss of antigen expression or upregulation of alternative immune checkpoints, including TIM-3 and LAG-3 (154). Therefore, the development of novel therapeutic approaches remains essential. For instance, the CheckMate 648 trial explored dual immune checkpoint blockade targeting both PD-1 and CTLA-4 in patients with ESCC. This strategy demonstrated significant efficacy even in the absence of chemotherapy, particularly among patients with high PD-L1 expression (133, 155). Another promising direction lies in adoptive immunotherapy, in which chemotherapy is used for initial tumor debulking, followed by the administration of tumor-infiltrating lymphocytes (TILs) or CAR-T cells engineered against ESCC-relevant antigens (156). Ultimately, the relationship between chemotherapy administration and the emergence of cellular resistance resembles a dynamic interplay, much like the evolutionary arms race between bacteria and antibiotics. While resistance cannot be entirely prevented, its onset can be delayed or mitigated through rational therapeutic strategies.
