# Correction: Overcoming chemoresistance in esophageal cancer with synergistic strategies

**DOI:** 10.3389/fimmu.2026.1917784

**Published:** 2026-07-03

**Authors:** Chunxiao Gao, Ting Zhang, Guangyuan Li, Weishan Zhang, Honglin Chen, Diefei Li, Jixuan Xu, Mengxue Li, Ruihong Wang, Yamin Xing, Jian Zhan, Zhentai Ren, Xia Xue, Wulong Liang, Xiangdong Sun, Xiaojuan Zhang, Xinfeng Liang, Hongqiao Zhan, Zisen Zhang, Xiufeng Chu

**Affiliations:** 1 Zhengzhou Key Laboratory of Lipid Metabolism and Precision Diagnosis & Treatment in Oncology, Oncology Department, the Fifth Affiliated Hospital of Zhengzhou University, Zhengzhou, China; 2 State Key Laboratory of Metabolic Dysregulation & Prevention and Treatment of Esophageal Cancer, Tianjian Laboratory of Advanced Biomedical Sciences, Zhengzhou, China; 3 Marshall B. J. Medical Center, The Fifth Affiliated Hospital of Zhengzhou University, Zhengzhou, China; 4 Department of Gastrointestinal & Thyroid Surgery, The Fifth Affiliated Hospital of Zhengzhou University, Zhengzhou, Henan, China; 5 Department of Respiratory and Critical Care Medicine, The Fifth Affiliated Hospital of Zhengzhou University, Zhengzhou, Henan, China; 6 Henan International Joint Laboratory of Glioma Metabolism and Microenvironment Research, The Fifth Affiliated Hospital of Zhengzhou University, Zhengzhou, Henan, China; 7 Scientific Research and Discipline Management Office, The Fifth Affiliated Hospital of Zhengzhou University, Zhengzhou, Henan, China

**Keywords:** chemoresistance, esophageal cancer, immunotherapy, targeted therapy, tumor microenvironment

**Supplemental Material [Supplementary Table 1]** was omitted. The file(s) have now been published.

In the published article, there was a mistake in the manuscript text. In Section 1 **Introduction**, the last sentence in the first paragraph was displayed as: “Furthermore, chemotherapy remains a fundamental component in of current combination therapies (5–7).” The correct sentence is: “Furthermore, chemotherapy remains a fundamental component of current combination therapies (5–7)”.

A correction has been made to the section In Section 1 **Introduction**, Paragraph 1:

“Since the initial efforts to use chemotherapeutic agents such as bleomycin, mitomycin C, and 5-fluorouracil (5-FU) for esophageal cancer treatment in the late 1960s, researchers have conducted extensive research to improve chemotherapy strategies (1, 2). For recurrent or metastatic esophageal squamous cell carcinoma (ESCC) and esophageal adenocarcinoma (EAC), first-line systemic therapy is commonly based on a fluoropyrimidine (5-FU or capecitabine) plus a platinum agent (3). Currently, chemotherapy has evolved its role from its initial use as a palliative attempt in advanced-stage patients to a cornerstone across the entire spectrum of esophageal cancer management. For locally advanced patients, neoadjuvant chemoradiotherapy or chemotherapy alone has become a standard strategy to enhance surgical resectability and improve survival rates. For those with recurrent or metastatic disease, chemotherapy remains the core palliative approach for alleviating symptoms and prolonging survival (3, 4). Furthermore, chemotherapy remains a fundamental component of current combination therapies (5–7).”

In Section 2.2.1 *Fibroblasts and immune cells*, the phrase “Immune Cells” at the beginning of the third paragraph was not formatted in bold. The phrase has now been corrected to bold formatting.

A correction has been made to the section In Section 2.2.1, Paragraph 3:

“**Immune Cells**: CD8+ T cells serve as the primary effector cells mediating antitumor immunity. In esophageal cancer, CD8+ T cells are not only numerically scarce but also functionally impaired owing to the elevated expression of immune checkpoint molecules, including PD-1, LAG-3, and TIM-3 (102, 103). In addition, M2- type tumor-associated macrophages (TAMs) suppress dendritic cells and effector T cells through the secretion of IL-10 and TGF- b (104). Regulatory T cells (Tregs) secrete IL-10 and TGF-b to suppress the functions of dendritic cells and effector T cells. Myeloid-derived suppressor cells (MDSCs) inhibit effector T cells through IL-6/STAT3-driven upregulation of arginase-1 and the production of reactive oxygen species (ROS) and inducible nitric oxide synthase (iNOS) (105, 106). For a more comprehensive overview of how different cells shape the tumor microenvironment, readers are referred to dedicated reviews on the TME (107–109).”

In Sections 2.1.1, in the last paragraph. The citation in parentheses incorrectly referred to “**Table 1**”. The correct citation is “**Supplementary Table 1**”.

A correction has been made to the section In Section 2.1.1, Paragraph 3:

“Efforts have been made to develop P-gp inhibitors to overcome drug efflux. The gut-specific inhibitor Encequidar (HM30181A) has completed a Phase III trial in breast cancer and demonstrated favorable safety and efficacy (NCT06835400). Meanwhile, another P-gp inhibitor, MB07133, is being evaluated in a Phase II trial for hepatocellular carcinoma (NCT00073736) (**Supplementary Table 1**). Although dedicated clinical trials of P-gp (or GST) inhibitors are currently lacking in esophageal cancer, the promising results from other malignancies suggest their potential applicability to overcoming chemoresistance in this setting (20, 32).”

In Sections 2.1.2, the last sentence in the last paragraph. The citation in parentheses incorrectly referred to “**Table 1**”. The correct citation is “**Supplementary Table 1**”.

A correction has been made to the section In Section 2.1.2, Paragraph 1:

“Platinum-based agents bind to DNA to form intra- or inter- strand crosslinks. This structural distortion induces severe DNA damage (10, 33). 5-Fluorouracil (5-FU) is an antimetabolite drug that mediates DNA and RNA damage through inhibition of thymidylate synthase and incorporation of its metabolites into DNA and RNA (34). Although DNA damage is intended to trigger tumor cell apoptosis, tumor cells counteract this effect through the DNA damage response (DDR) system (33, 35, 36). Key repair pathways include homologous recombination repair (HRR), non- homologous end joining (NHEJ), and nucleotide excision repair (NER). In esophageal cancer, elevated expression of crucial DDR proteins, such as RAD51 (in HRR) (37), DNA-PK and Ku70/80 (in NHEJ) (38, 39), and ERCC1 (in NER) (40), is closely associated with resistance to chemotherapy. Given the pivotal role of DDR in chemoresistance, inhibitors against key DDR regulators are under active development (41, 42). A prime example is the use of PARP inhibitors (e.g., Olaparib, Niraparib) (43). In tumor cells with a pre- existing HRR defect, PARP inhibition further disrupts the repair of single-strand breaks (SSBs). This dual impairment of both major repair pathways leads to an accumulation of lethal DNA damage, a mechanism known as synthetic lethality (41, 42). In esophageal cancer, combining PARP inhibitors with DNA-damaging chemo- therapy is being actively explored. The rationale is to use chemo- therapy to create DNA damage while employing a PARP inhibitor to block its repair, thereby synergistically enhancing tumor cell killing. This approach is particularly effective for esophageal cancer harboring homologous recombination deficiency. Corresponding clinical trials are underway, including studies evaluating Olaparib (NCT01460888) and Niraparib (NCT03840967) in esophageal/gastroesophageal cancers (**Supplementary Table 1**).”

In Sections 2.1.3, in the second paragraph. The citation in parentheses incorrectly referred to “**Table 1**”. The correct citation is “**Supplementary Table 1**”.

A correction has been made to the section In Section 2.1.3, Paragraph 2:

“To overcome cell cycle-related chemoresistance, CDK4/6 inhibitors have been developed. Among them, Palbociclib (Ibrance), Ribociclib (Kisqali), and Abemaciclib (Verzenio) are already ap- proved for clinical use in breast cancer. In esophageal cancer, PD- 0332991 and SHR6390 have demonstrated efficacy in suppressing tumor growth in preclinical experiments (48, 49). Moreover, a phase II clinical trial led by the Abramson Cancer Center to evaluate Palbociclib in advanced esophageal and gastroesophageal cancers (NCT01037790) is underway (**Supplementary Table 1**).”

In Sections 2.1.3, in the fourth paragraph. The citation in parentheses incorrectly referred to “**Table 1**”. The correct citation is “**Supplementary Table 1**”.

A correction has been made to the section In Section 2.1.3, Paragraph 4:

“To overcome apoptosis-related chemoresistance, BCL-2 inhibitor AT-101 has been found to enhance the cytotoxic effects of chemotherapeutic agents by promoting apoptosis in esophageal cancer (53). In addition, BCL-2/BCL-xL inhibitor TW37, when combined with the P-gp inhibitor verapamil, effectively increases the sensitivity of esophageal cancer to paclitaxel (32). Furthermore, the frequent loss of p53 function in esophageal cancer enables tumor cells to evade apoptosis induction (54, 55). Restoring p53 function has therefore emerged as another strategy to re-sensitize tumors. For instance, APR-246 (also known as eprenetapopt, a first- in-class small-molecule reactivator of mutant p53 currently under clinical investigation) (56), in combination with 5-FU, has demonstrated synergistic induction of apoptosis in preclinical esophageal cancer models (57, 58). Notably, this combination is also being evaluated clinically (NCT02999893) (**Supplementary Table 1**).”

The original version of this article has been updated.

