# Supplementary material for: Overcoming chemoresistance in esophageal cancer with synergistic strategies
Source: Front Immunol. 2026 Jun 3;17:1784325. doi: 10.3389/fimmu.2026.1784325 (PMC13272350; doi:10.3389/fimmu.2026.1784325)
Supplement: Supplementary file 1 [file Table1.docx]

**Supplementary Table 1. Advances in chemoresistance-targeted therapy in esophageal cancer and other cancers.**

| ****Trial ID**** | Treatment | ****Cancer type**** | ****Phase**** | Ages Eligible | Sexes Eligible for Study | Study Start | Study Completion | Enrollment | Interventional Model |
| --- | --- | --- | --- | --- | --- | --- | --- | --- | --- |
| NCT01460888 | *Olaparib + radical radiotherapy (ROCOCO)* | Esophageal cancer | I | 18 Years and older | All | 2013-07 | 2018-08 | 36 | Parallel Assignment |
| NCT03840967 | Niraparib | Esophageal/GEJ/  proximal gastric adenocarcinoma | II | 18 Years and older | All | 2019-07 | 2023-02 | 14 | Single Group Assignment |
| NCT01037790 | Palbociclib  （PD-0332991） | Refractory solid tumors | II | 18 Years and older | All | 2009-10 | 2019-10 | 304 | Single Group Assignment |
| NCT02999893 | APR-246 + cisplatin  + 5-FU | Esophageal/GEJ cancer, advanced/metastatic, platinum-resistant | I/II | 18 Years and older | All | 2017-04 | 2021-02 | 5 | Single Group Assignment |
| NCT06835400 | Oral paclitaxel  + encequidar | Breast Cancer HER2(-), Metastatic | III | 18 Years and older | All | 2025-09 | 2029-05 | 340 | Parallel Assignment |
| NCT00073736 | MB07133 | HCC  unresectable | I/II | 18 Years and older | All | 2003-09 | 2007-07 | 28 | Single Group Assignment |

***Notes:****Many drug resistance mechanisms are found in different cancers, including esophageal cancer. Therefore, drugs studied in other cancers were also included in this table because they may*

*have potential in esophageal cancer.*
